# Successful non-operative management of wound dehiscence after PSARP: case report

**DOI:** 10.3389/fped.2026.1933431

**Published:** 2026-10-29

**Authors:** Fatimah Almabyouq, Zahrh Faisal Abualsaud, Walaa Salman Alsharrah

**Affiliations:** Department of Pediatric Surgery, Imam Abdulrahman Bin Faisal University King Fahad Hospital of the University, Al Khobar, Saudi Arabia

**Keywords:** anorectal malformation (ARM), anterior displacement of anus, conservative management, hemoglobin spray, posterior sagittal anorectopelasty (PSARP), Silvercel dressing, wound dehiscence

## Abstract

**Background:**

Wound dehiscence is a common early postoperative complication following Posterior Sagittal Anorectoplasty (PSARP) for Anorectal malformation (ARM). Conservative wound management is an effective strategy to promote healing while preserving neoanus function after postoperative complications. We present a case of wound dehiscence following PSARP for ARM, treated conservatively with an excellent outcome and preservation of neoanus function.

**Case presentation:**

We present a preterm female neonate with a rectoperineal fistula and cardiac anomalies who underwent PSARP at four months of age. The patient developed wound dehiscence with a 5-mm gap at perineal body and foul-smelling discharge on the fifth postoperative day. Conservative management was implemented with consistent dressings containing Silvercel and a hemoglobin-based spray. This was done because the parents refused surgical intervention in the form of stoma creation. This approach resulted in substantial wound improvement and preservation of the neoanus and sphincter function.

**Conclusion:**

This case suggests that conservative, multidisciplinary management may be a feasible option for selected cases of superficial wound dehiscence after PSARP when the neoanus is intact and the patient is clinically stable. Topical purified hemoglobin spray with advanced dressings was associated with progressive wound healing and preserved neoanal function. Further pediatric studies are needed to confirm its safety and effectiveness for post-PSARP wound complications.

## Introduction

Anorectal malformations (ARMs) comprise a heterogeneous group of congenital anomalies that require individualized surgical reconstruction to establish an anatomically positioned, functional anus. Posterior sagittal anorectoplasty (PSARP) is a widely used definitive approach because it permits direct identification of the rectum and sphincter complex; however, perineal wound complications remain an important source of postoperative morbidity (1, 3). Wound dehiscence may range from limited separation at the anocutaneous junction to deeper disruption involving the reconstructed perineal body and muscle complex, with potential consequences for infection, neoanal integrity, and long-term continence (1, 4).

Management depends on the depth of dehiscence, the condition of the neoanus, the presence of infection, and the patient's overall clinical stability. Fecal diversion is often considered when contamination or deeper tissue disruption threatens the repair, and a divided colostomy may reduce exposure of the perineal wound to enteric organisms (1, 4). In selected superficial cases, however, meticulous local wound care, nutritional support, antimicrobial dressings, and close multidisciplinary assessment may allow healing without reoperation. Evidence guiding this approach in infants remains limited.

## Case presentation

A female neonate was born at 36 + 6 weeks of gestation via elective cesarean section to a 41-year-old mother with a history of four prior cesarean sections. The antenatal course was uncomplicated, except for gestational diabetes mellitus, which was managed with oral hypoglycemics. The anomaly scan showed no significant findings. The neonate weighed 2.9 kg at birth.

As an infant of a diabetic mother (IDM), she was admitted to the NICU for observation. Physical examination revealed significant findings, including collodion skin, left-hand polydactyly, and a rectoperineal fistula. Echocardiography showed a small patent foramen ovale (PFO) and a patent ductus arteriosus (PDA). Based on the constellation of anomalies, a diagnosis of VACTERL association was confirmed.

The Pediatric Surgery service was consulted to assess the Recto-Perineal Fistula, and serial anal dilation was started on the second day of life with Hegar dilators, beginning at size 6 and advancing to size 8.5 by the fifth day. The patient was discharged on the sixth day of life with instructions for the mother to continue dilation at size 8. Consistent clinic follow-up facilitated ongoing advancement of dilatation, using sizes 7 to 9, in anticipation of definitive surgical intervention. Other VACTERL association findings were managed according to the appropriate specialty.

At four months of age and weighing 6.6 kg, the patient was admitted electively under the care of Pediatric Surgery and prepared with NPO and a rectal wash with 400 mL of saline. Prophylactic antibiotics [Amoxicillin/Clavulanic Acid and Metronidazole] were given according to protocol. She underwent a Formal Posterior Sagittal Anorectoplasty (PSARP). The procedure went smoothly with no complications.

Postoperatively, she was transferred back to the pediatric surgical ward. Antibiotics were continued for 48 h with appropriate analgesia. Local wound care was performed with Fucidin, Lidocaine, and Miconazole creams.

On the third day postoperatively, the wound showed mild erythema around the perineal body, with no discharge noted and the neoanus intact. Irrigation was performed, and Fucidine was applied. On day 5 postoperatively, superficial wound dehiscence of approximately 0.5 cm on the perineal body was noted, with foul-smelling discharge and spontaneous detachment of Monocryl sutures, suggesting a potential regional allergic reaction to the sutures. The neoanus was intact (Figure 1).

**Figure 1:**
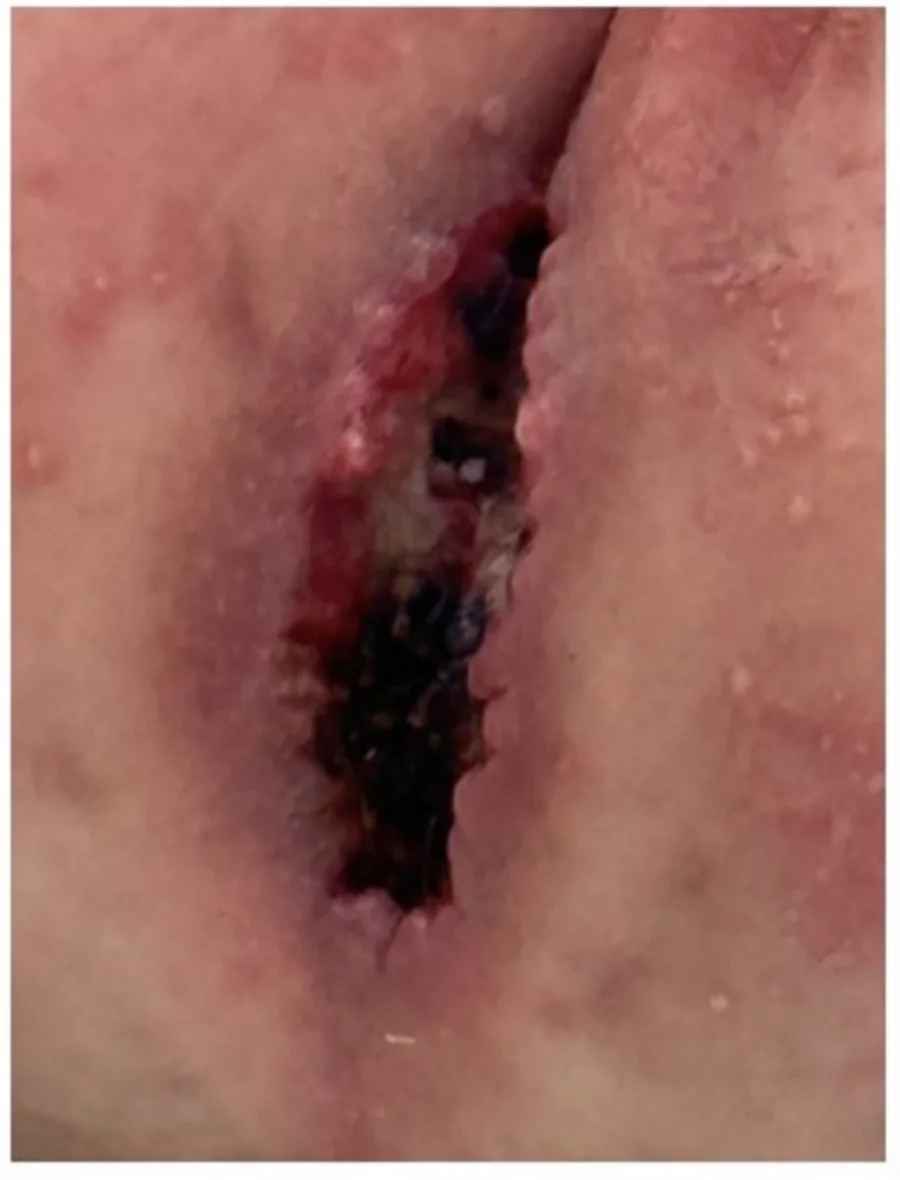
Focused view on the wound status on postoperative day 5.

A multidisciplinary team was formed. The wound care team was contacted to assess the wound. Dietitian and clinical pharmacology services were contacted to initiate Total Parenteral Nutrition (TPN). A Peripherally Inserted Central (PICC) Line was inserted. The dermatology team was contacted to treat diaper rash, and General Pediatrics was consulted for optimization. Both parents were consulted regarding colostomy creation to preserve sphincter function, prevent possible future incontinence, and control infection. They strongly refused.

The patient was kept NPO for a total of one month, and TPN was started uneventfully. Dressings with Granulox (Hemoglobin-based spray, Molnlycke, Sweden) and Silvercel (Exofiber Ag+, Molnlycke, Sweden) were performed every other day for a period of one month. Intermittent minimal stimulation of new granulation tissue was performed with wet gauze to promote recovery. The patient showed marked improvement with an excellent wound response.

Anal dilation of the neoanus started one month postoperatively, with good contractility and stool continence. Hemoglobin spray was continued with Silvercel dressing every 3 days for 2 weeks, with a 1-week gap using wet-to-dry gauze dressing. She was discharged 5 weeks after the procedure with wound care follow-up and clinic appointments.

Patient was seen 7 weeks after the procedure in the pediatric surgery clinic with no active complaints. Mother started introducing semisolid foods, and the patient was passing bowel movements regularly, with preserved anal function and no stool incontinence. The wound was healing well (Figures 2A,B).

**Figure 2:**
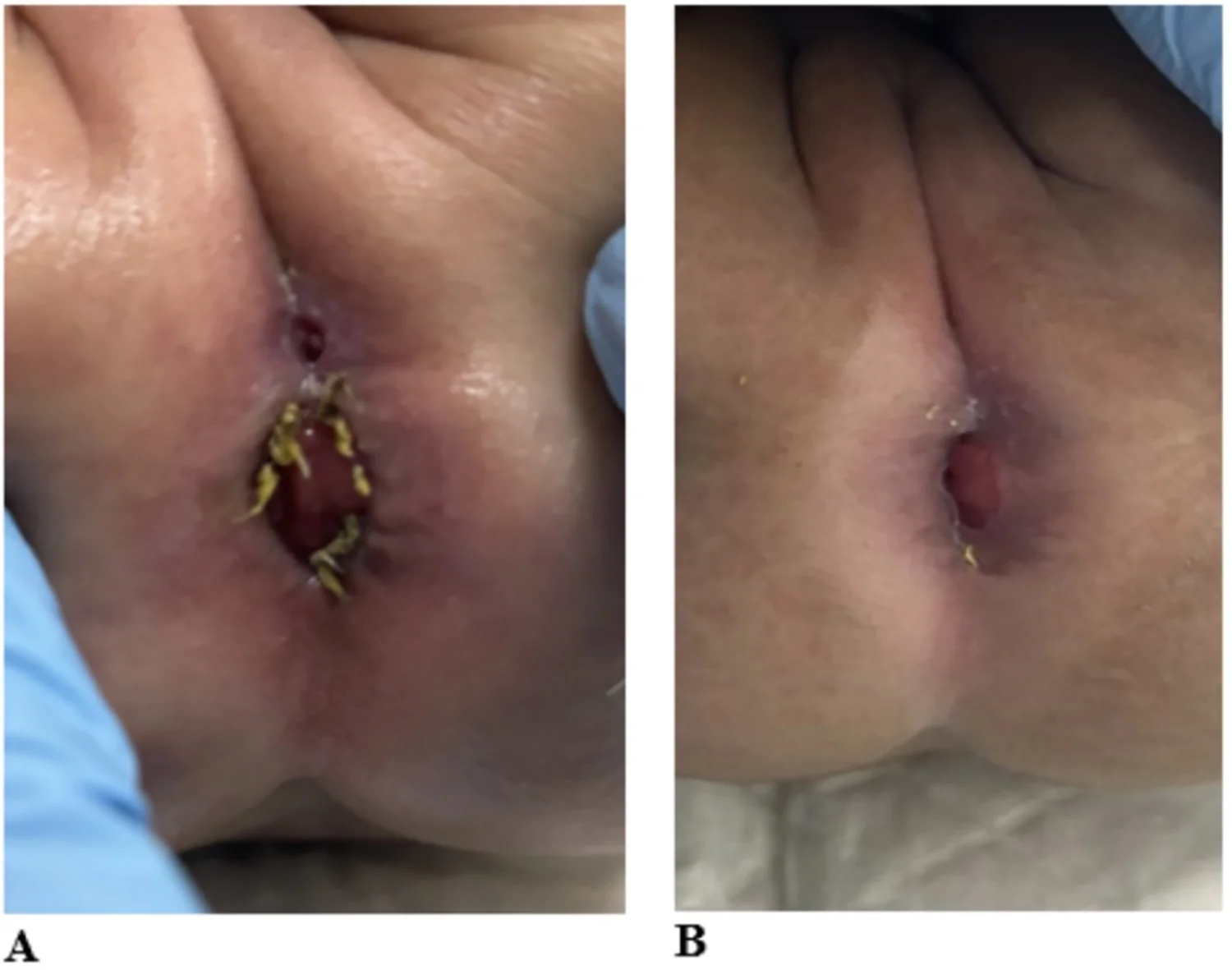
**(A)** wound status 7 weeks postoperatively. Good granulaton noted with intact Neoanus. Sutures still attached with new skin bridge noted. **(B)** Wound Status 9 weeks postoperatively. Marked improvement in Ano-cutaneous healing with intact sensation and good anal tone and functional continence. Mild mucosal prolapse noted.

When she reached 9 months of age, Mother reported episodes of constipation that were treated with a Glycerin suppository. The neoanus was continent, with preservation of function (Figure 3).

**Figure 3:**
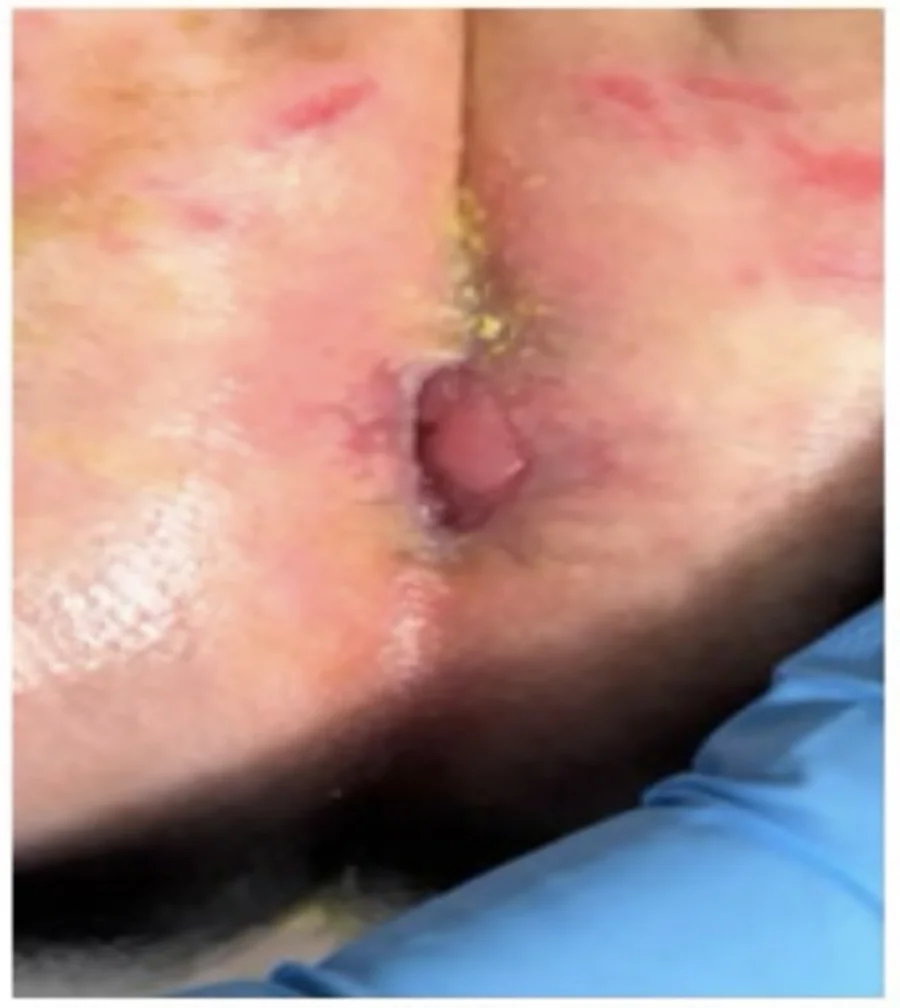
Wound status 3 months postoperatively with good intact function.

Patient continued to follow up at our clinic annually, reached 2 years of age, and remained symptom-free. Toilet training started without issues.

## Discussion

Posterior Sagittal Anorectoplasty (PSARP) is the most common surgical repair for anorectal malformation (ARM) (1). Wound dehiscence is a common early postoperative complication and can be classified as superficial [involving the anocutaneous junction] or deep [including disruption of repaired muscles] (1).

The pathogenesis of post-PSARP wound dehiscence is not fully established, but bacterial infection and decreased tissue perfusion have been suggested as contributing factors (2, 3).

In addition, the type of surgery (single- or multi-staged procedure), gender, age at repair, prematurity, birth weight, type of ARM, associated anomalies, and use of antibiotics (1). During the literature review, it was found that the presence of cardiac anomalies is a significant risk factor for wound healing (1). Wound dehiscence is less likely to occur in multi-staged PSARP with a divided colostomy (1, 4). The colostomy has previously been acknowledged to protect the surgical site after PSARP from postoperative infections (1). Theoretically, this could be because the colostomy prevents fecal bacteria from entering the perineal wound.

To reduce the risk of post-PSARP wound complications, various strategies have been proposed, including prolonged intravenous antibiotic prophylaxis, topical antibiotics, standardized wound cleansing, preoperative bowel irrigation, postoperative fasting, and vacuum-assisted wound closure (2).

Furthermore, some authors suggest that proper hemostasis and gentle handling of tissues play an important role in avoiding wound dehiscence. In addition, the use of monofilament sutures will reduce surgical site infection (1). On the other hand, extensive dissection, rough tissue handling, and excessive diathermy cause tissue damage and hematoma formation, which lead to surgical site infection and poor wound healing. They might also cause retraction of the neo-anus and/or perineal body disruption, which mandates redo surgical repair and will affect the final outcome and continence (1).

Some modified surgical techniques might prevent wound dehiscence, such as a novel Anoplasty technique employing a Para U-Stitch along the anterior and posterior portions of the Anoplasty, which buttresses the midline U-stitch and everts the rectal mucosa (5), and postoperative application of VAC therapy over the Perineal Wound to prevent wound dehiscence of the neoanus and reconstructed perineal body (6).

In our case, the patient's parents refused the surgical option of stoma creation. The clinical finding of spontaneous detachment of Monocryl sutures may suggest a questionable allergic reaction to Monocryl sutures, as documented adult cases of rare skin reactions to poliglecaprone 25 or glyconate sutures exist, and adverse reactions to other types of sutures, such as polyglactin sutures, have also been reported (7). The mother reported an incident of wound dehiscence in her cesarean section scar in the first week postoperatively.

Several methods have been used to promote appropriate and timely wound healing. The role of oxygen in healing is not fully understood. In the literature, some studies have demonstrated the effectiveness of hyperbaric oxygen therapy chambers in healing chronic wounds and treating several medical conditions, such as carbon monoxide poisoning and necrotizing fasciitis. Dr. Figen Aydin showed that hyperbaric oxygen treatment can be life- or limb-saving and is a safe treatment with a low risk of adverse events in pediatric patients (8).

On the other hand, Copeland et al. showed that complicated sternal wound infections after pediatric cardiac surgery that are refractory to antibiotic therapy and/or debridement can be successfully treated with Negative Pressure Wound Therapy and/or Hyperbaric Oxygen Therapy (9). Unfortunately, the application of Hyperbaric therapy has been limited due to restricted access and the difficulty of implementation in real-world settings.

The Topical 99.9% purified hemoglobin spray is a method available for wound care. It accelerates wound healing by increasing oxygenation at the wound surface through facilitated diffusion. Purified hemoglobin spray has been used for wounds with prolonged healing, including burns, venous ulcers, diabetic foot ulcers, infected post-traumatic wounds, and postoperative wounds, with studies reporting that it can accelerate the healing process (10–13). Purified hemoglobin spray may also increase collagen synthesis by promoting the hydroxylation of proline and lysine, thereby contributing to the formation of a stable collagen structure (14).

Exufiber Ag ^+^ is a gelling fiber dressing that releases ionic silver (Ag⁺) when exposed to wound exudate. The Ag⁺ ions provide broad-spectrum antimicrobial activity by disrupting microbial cell membranes, inhibiting key metabolic enzymes, and interfering with DNA replication, resulting in effective reduction of wound bioburden. Its Hydrolock® fiber structure absorbs and retains exudate while maintaining dressing integrity, helping create a cleaner wound environment that supports healing (15).

In the pediatric population, data on the use of purified hemoglobin spray are limited. Kulice demonstrated its effectiveness in the first published study of pediatric patients with second-degree burns (14). The promising results might open a new path for future research.

## Conclusion

This case suggests that conservative, multidisciplinary management may be a feasible option for selected cases of superficial wound dehiscence after PSARP when the neoanus is intact and the patient is clinically stable. Topical purified hemoglobin spray with advanced dressings was associated with progressive wound healing and preserved neoanal function. Further pediatric studies are needed to confirm its safety and effectiveness for post-PSARP wound complications.

## Data Availability

The datasets presented in this article are not readily available because of ethical and privacy restrictions. Requests to access the datasets should be directed to the corresponding authors.
